# Met carriers of the *BDNF* Val66Met polymorphism show reduced Glx/NAA in the pregenual ACC in two independent cohorts

**DOI:** 10.1038/s41598-021-86220-3

**Published:** 2021-03-24

**Authors:** Louise Martens, Luisa Herrmann, Lejla Colic, Meng Li, Anni Richter, Gusalija Behnisch, Oliver Stork, Constanze Seidenbecher, Björn H. Schott, Martin Walter

**Affiliations:** 1 University Department of Psychiatry and Psychotherapy, Tübingen, Germany; 2 Graduate Training Center, IMPRS, Tübingen, Germany; 3 Max Planck Institute for Biological Cybernetics, Tübingen, Germany; 4 Department of Psychiatry and Psychotherapy, Jena University Hospital, Jena, Germany; 5 Clinical Affective Neuroscience Laboratory, Magdeburg, Germany; 6 Department of Psychiatry, Yale School of Medicine, New Haven, CT USA; 7 Leibniz Institute for Neurobiology, Magdeburg, Germany; 8 Department of Genetics & Molecular Neurobiology, Institute of Biology, Otto-Von-Guericke-University, Magdeburg, Germany; 9 Center for Behavioral Brain Sciences, Magdeburg, Germany; 10 Department of Psychiatry and Psychotherapy, University Medicine Göttingen, Göttingen, Germany; 11 German Center for Neurodegenerative Diseases (DZNE), Göttingen, Germany

**Keywords:** Genetics, Neuroscience, Medical research, Risk factors

## Abstract

The Met allele of the Val66Met SNP of the *BDNF* gene (rs6265) is associated with impaired activity-dependent release of brain-derived neurotrophic factor (BDNF), resulting in reduced synaptic plasticity, impaired glutamatergic neurotransmission, and morphological changes. While previous work has demonstrated Val66Met effects on magnetic resonance spectroscopy (MRS) markers of either glutamatergic metabolism (Glx) or neuronal integrity (NAA), no study has investigated Val66Met effects on these related processes simultaneously. As these metabolites share a metabolic pathway, the Glx/NAA ratio may be a more sensitive marker of changes associated with the Val66Met SNP. This ratio is increased in psychiatric disorders linked to decreased functioning in the anterior cingulate cortex (ACC). In this study, we investigated the correlation of the Val66Met polymorphism of the *BDNF* gene with Glx/NAA in the pregenual anterior cingulate cortex (pgACC) using MRS at 3 Tesla (T) (n = 30, all males) and 7 T (n = 98, 40 females). In both cohorts, Met carriers had lower Glx/NAA compared to Val homozygotes. Follow-up analyses using absolute quantification revealed that the Met carriers do not show decreased pgACC glutamate or glutamine levels, but instead show increased NAA compared to the Val homozygotes. This finding may in part explain conflicting evidence for Val66Met as a risk factor for developing psychiatric illnesses.

## Introduction

The brain-derived neurotrophic factor (*BDNF*) is a key molecule in neural plasticity that is involved in neuronal cell growth and differentiation, but also apoptosis^1,2^. In the mature synapse, it plays an important role in neurotransmission and synaptic plasticity^3,4^. In humans, a common single-nucleotide polymorphism (SNP) of the *BDNF* gene on chromosome 11p14.1 (196 G > A) results in a substitution of valine (Val) with methionine (Met) at codon 66 (Val66Met; NCBI accession number: rs6265). The Val66Met polymorphism has attracted considerable attention in psychiatric research, as the 66Met allele impairs intracellular trafficking and (activity-dependent) release of BDNF into the synaptic cleft^5,6^, even in heterozygous carriers^7^. The polymorphism is associated with the course and development of various neurological and psychiatric disorders^8–10^.

BDNF release is tightly linked to glutamatergic neurotransmission. BDNF facilitates glutamate release, influences excitatory synapse formation, and increases NMDA receptor and AMPA receptor subunit phosphorylation. These processes enhance long-term potentiation (LTP)^11^. Presynaptically, BDNF signaling regulates glutamate release^12,13^. At the postsynaptic side, BDNF enhances the expression of late-phase LTP by promoting gene expression, protein synthesis, and the availability of glutamate receptors^11,14,15^. The activity-dependent release of BDNF from postsynaptic glutamatergic terminals is specifically impaired by the Val to Met substitution^6,16^, which interferes with intracellular sorting of BDNF^7^. As a result, glutamatergic synapse function is impaired in the rodent prefrontal cortex (PFC) and hippocampus^17,18^. In humans, the Met allele is associated with reduced hippocampal and prefrontal volume^19–22^. This suggests that gross morphological changes related to *BDNF* Val66Met can be detected with magnetic resonance imaging (MRI) in humans.

As Val66Met affects both glutamatergic neurotransmission and synaptic structure, its effects may be better characterized when both these targets are assessed simultaneously. One way to do so is by means of magnetic resonance spectroscopy (MRS). MRS can non-invasively quantify local in vivo concentrations of metabolites such as N-acetylaspartate (NAA) and Glx. NAA is a mitochondrial metabolite often reported as a proxy for neuronal integrity and viability^23–25^.Glx represents the sum of glutamate and glutamine concentrations. Previous work has studied NAA or Glx separately. Left hippocampal Glx (Met homozygotes only^26^) and NAA were reduced in Met carriers^6,26,27^. The distribution of metabolites like NAA-as well as Glx-as a function of *BDNF* Val66Met genotype may be subject to regional differences, as in the anterior cingulate cortex (ACC) NAA levels are increased in Met carriers^28^. Given the shared metabolic pathways of these molecules^29,30^ and the correlation of these metabolites often reported in healthy participants^31–33^, the ratio of Glx/NAA may be a more sensitive marker of dysregulation due to Val66Met.

The Val66Met polymorphism has been associated with psychiatric disorders like schizophrenia or major depressive disorder (MDD), either as a risk factor or as a protective factor^8,34–38^. In both disorders, decreases in gray matter volume have been reported for the ACC, especially for the pregenual part (pgACC)^39–42^. Since decreases in hippocampal and prefrontal gray matter volume have been shown for the Met allele of the Val66Met SNP, a reduced gray matter volume in the pgACC might be linked to the Val66Met SNP as well. The pgACC is a region implicated in the pathophysiology of several psychiatric disorders^43^, but that has not been investigated in the context of the Val66Met SNP. Investigating Val66Met effects on the Glx/NAA ratio in the pgACC could therefore provide insight into downstream functional consequences of the polymorphism that could indicate the Met allele as a protective or a risk factor.

Here, we investigate the effects of the *BDNF* Val66Met polymorphism on an index of glutamatergic functioning and neuronal integrity in the pgACC. To this end, we used MRS to measure pgACC levels of Glx as a ratio over NAA. For a more robust assessment of the effect of the Val66Met SNP, two independent cohorts were investigated at 3 T and 7 T, respectively. We hypothesized that because the Val66Met polymorphism affects neurodevelopment as well as glutamatergic neurotransmission, Met carriers would show lower Glx/NAA compared to Val homozygotes. We expected that this would be primarily due to reduced concentrations of glutamate-related metabolites since specifically in the pgACC a decrease in Glx has been associated with MDD^44,45^. A decrease in NAA, on the other hand, has been associated with schizophrenia^46^. The increase in NAA in the ACC has been suggested to be just a compensatory mechanism for a deficit in BDNF regulation speaking for a decreased risk to develop schizophrenia and for a deficit in hippocampal NAA^6,27,28^. We tested this hypothesis in the second cohort, where the higher field strength of 7 T allowed us to perform exploratory analyses of absolute concentrations of NAA, as well as the major contributors of Glx (glutamate + glutamine), considering their better separation at ultra-high field.

## Results

### Demographics

Descriptive statistics of the sample used in the main analyses are summarized in Table 1. Allele frequencies of both datasets were at Hardy–Weinberg equilibrium (cohort 1: N = 30, χ^2^ = 0.016, *P* = 0.899, D = 0.408, cohort 2: N = 98, χ^2^ = 2.110, *P* = 0.146, D = 2.296).

**Table 1:** Demographic characteristics of *BDNF* Val66Met Met carriers and Val homozygotes.

	Cohort 1			Cohort 2			Cohort comparison
	Met carrier	Val/Val	Statistics	Met carrier	Val/Val	Statistics	Statistics
N	10	17	–	30	68	–	χ^2^ = 0.16, *P* = .69
Age	28.30 ± 3.34	30.65 ± 6.08	*t*(24.950) = − 1.29, *P* = .21	25.83 ± 4.98	27.00 ± 6.90	t(75.28) = − 0.94, *P* = .35	*p*_*boot*_ = .01
Gender	10 m	17 m	-	17 m / 13 f	41 m / 27 f	χ^2^ = 0.01, *P* = .91	-
GM ratio	0.72 ± 0.02	0.72 ± 0.03	*t*(22.89) = 0.46, *P* = .65	0.75 ± 0.05	0.76 ± 0.05	t(61.95) = − 0.65, *P* = .52	*p*_*boot*_ < .001

GM ratio: ratio of gray matter volume in the pgACC voxel. Between-cohort comparisons of age and GM ratio with bootstrapped one-sample t-tests (20,000 iterations).

### Decreased Glx/NAA in Met carriers of the Val66Met polymorphism

#### Cohort 1

**Figure 1:**
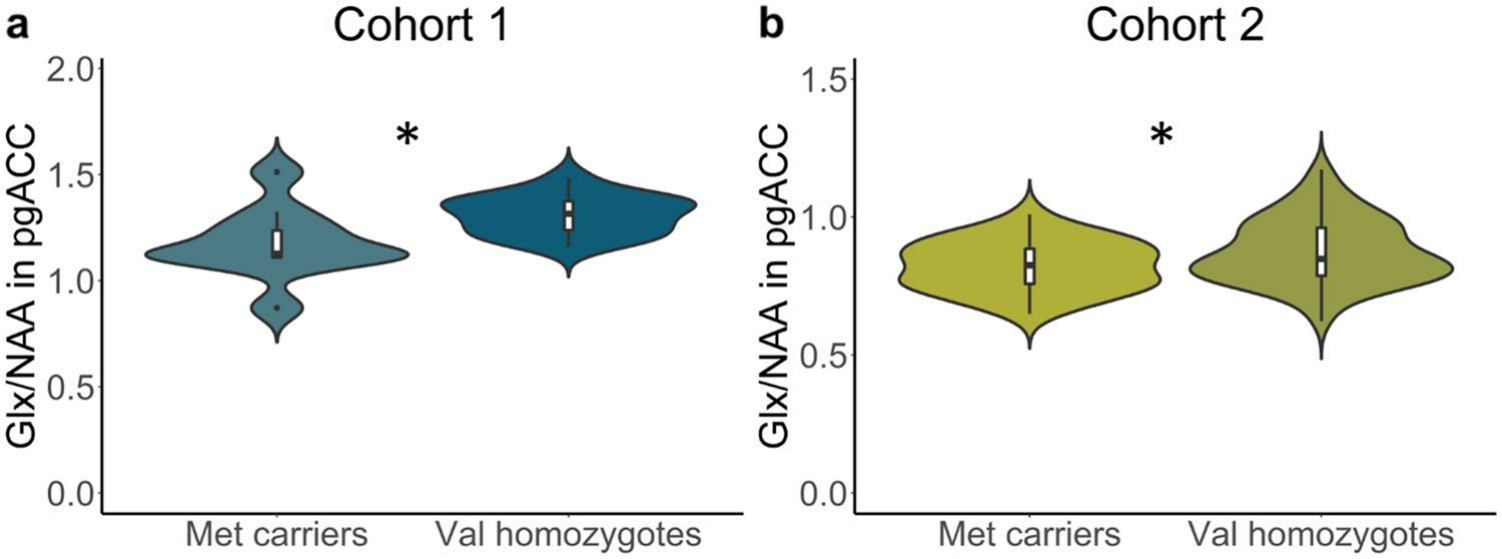
*BDNF* Val66Met effects on pgACC Glx/NAA. Violin plots represent distributions of Glx/NAA values corrected for the effects of confounders (cohort 1: age, gray matter tissue proportion; cohort 2: age, gray matter tissue proportion and gender). (**a**) Results of cohort 1 (3 T, n = 26). (**b**) Results of cohort 2 (7 T, n = 97). *P < .05.

To test whether *BDNF* Val66Met carriers had altered Glx/NAA compared to Val homozygotes, we performed a linear model analysis with Glx/NAA as the outcome variable and *BDNF* genotype as predictor, controlling for the effect of age and gray matter proportion in the MRS voxel. Data from one participant was classified as an outlier and removed (see *Methods, Statistical analysis*). The assumption of normality of model residuals was met (Shapiro-Wilke, *P* < 0.05). The linear model revealed a significant effect of *BDNF* genotype, *F*(1,22) = 6.756, *P* = 0.016, *η*^2^_partial_ = 0.230 (Fig. 1a). There was no significant effect of age (*P* = 0.737) or gray matter proportion (*P* = 0.093). When testing for the directionality of the *BDNF* genotype effect, a t-test for unequal variances revealed that the Glx/NAA ratio was higher in Val homozygotes (M = 1.310, SD = 0.095) compared to Met carriers (M = 1.176, SD = 0.179), *t* (12.246) = − 2.191, *P* = 0.049, 95% CI [− 0.268, − 0.001].

#### Cohort 2

To test whether reduced Glx/NAA could be reproduced in a larger cohort measured at the ultra-high field strength of 7 T, we performed a linear model analysis as described above, additionally controlling for gender since metabolite concentrations may vary as a function of sex^47^. We excluded one data point that was classified as an outlier (see *Methods, Statistical analysis*). Model residuals were normally distributed (Shapiro-Wilke, *P* < 0.05). There was a significant effect of *BDNF* genotype on pgACC Glx/NAA, *F*(1,92) = 4.597, *P* = 0.035, *η*^2^_partial_ = 0.050 (Fig. 1b). There were no significant main effects of age (*P* = 0.411) and pgACC gray matter proportion (*P* = 0.384). We observed a significant main effect of gender (*P* = 0.026). To further explore this, we ran unplanned follow-up analysis adding the interaction term *BDNF* group*gender to the model. This revealed no significant interaction effect, *P* = 0.999.

Analogous to the analysis of cohort 1, we performed a t-test of unequal variances to test for the directionality of the main effect of *BDNF* genotype. This revealed that Val homozygotes had significantly higher Glx/NAA (M = 0.907, SD = 0.122) compared to Met carriers (M = 0.853, SD = 0.093), *t*(71.593) = − 2.394, *P* = 0.019, 95% CI [− 0.099, − 0.009].

### Converging evidence for a reduction of Glx/NAA in Met carriers of the Val66Met polymorphism

Combining the *P*-values of the main effect of *BDNF* genotype on pgACC Glx/NAA from two independent cohorts using Fisher’s method revealed converging evidence for a reduction of pregenual Glx/NAA in Met carriers, *χ*^2^(4) =14.943, *P* = 0.005.

### Increased NAA only in male Met carriers of the Val66Met polymorphism

To tease apart the *BDNF* Val66Met effects on aspects of excitatory neurotransmission and proxies of neuronal integrity, we capitalized on the increased signal dispersion at ultra-high field strength of 7 T in cohort 2. In post-hoc analyses, we performed absolute quantification of NAA, Glu, and Gln and modeled *BDNF* effects on these metabolites separately, controlling for the influence of age and gender. Voxel tissue composition was taken into account during the absolute quantification procedure (see Materials and Methods). For all analyses reported in this section, the assumption of normality of residuals was met (Shapiro-Wilke, *P* < 0.05).

**Figure 2:**
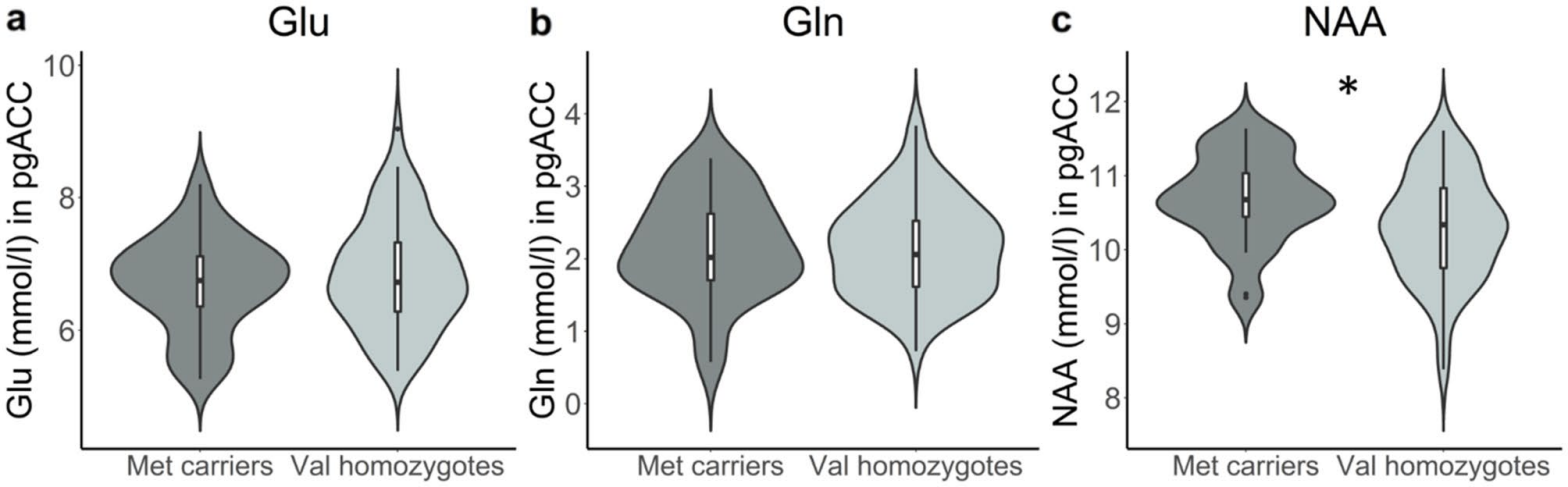
Absolute quantification. Results of follow-up linear regression models for (**a**) Glutamate, (**b**) Glutamine, and (**c**) NAA, with *BDNF* Val66Met genotype (Met homozygote or Val carrier) as predictor, controlling for age and gender. Violin plots show the distribution of metabolite values, uncorrected for covariates. *P < .05 after Bonferroni correction for multiple comparisons. *Mmol/l* millimolar; *Glu* glutamate; *Gln* glutamine; *NAA* N-acetylaspartate.

For the analysis of Gln, four data points were removed because of insufficient data quality. For the analysis of both NAA and Gln, four data points were classified as outliers and subsequently removed. For the analysis of Glu, two outliers were removed. Glu concentration revealed no significant main effect of genotype (*F*(1,92) = 0.606, *P* = 0.438, *η*^2^_partial_ = 0.004, Fig. 2a), age (*F*(1,92) = 0.696, *P* = 0.406) or gender (*F*(1,92) = 2.261, *P* = 0.136). Gln concentration showed no main effect of genotype (*F*(1,86) = 0.016, *P* = 0.901, *η*^2^_partial_ < 0.001, Fig. 2b) or age (*F*(1,86) = 0.071, *P* = 0.790), but a significant main effect of gender, *F*(1, 86) = 6.159), *P* = 0.015, *η*^2^_partial_ = 0.067), such that male participants had higher Gln concentrations (M = 2.521, SD = 0.692) compared to female participants (M = 2.153, SD = 0.640). Including the interaction term of genotype*gender in the model revealed no significant interaction effect (*F*(1,85) = 0.308, *P* = 0.581).

Most interestingly, the *BDNF* genotype was significantly associated with NAA levels in the pgACC, *F*(1,90) = 6.356, *P* = 0.013, *η*^2^_partial_ = 0.068 (Fig. 2c), such that Met carriers had higher NAA (M = 10.448, SD = 0.541) compared to Val homozygotes (M = 10.050, SD = 0.766). This result remained significant after Bonferroni correction.

**Figure 3:**
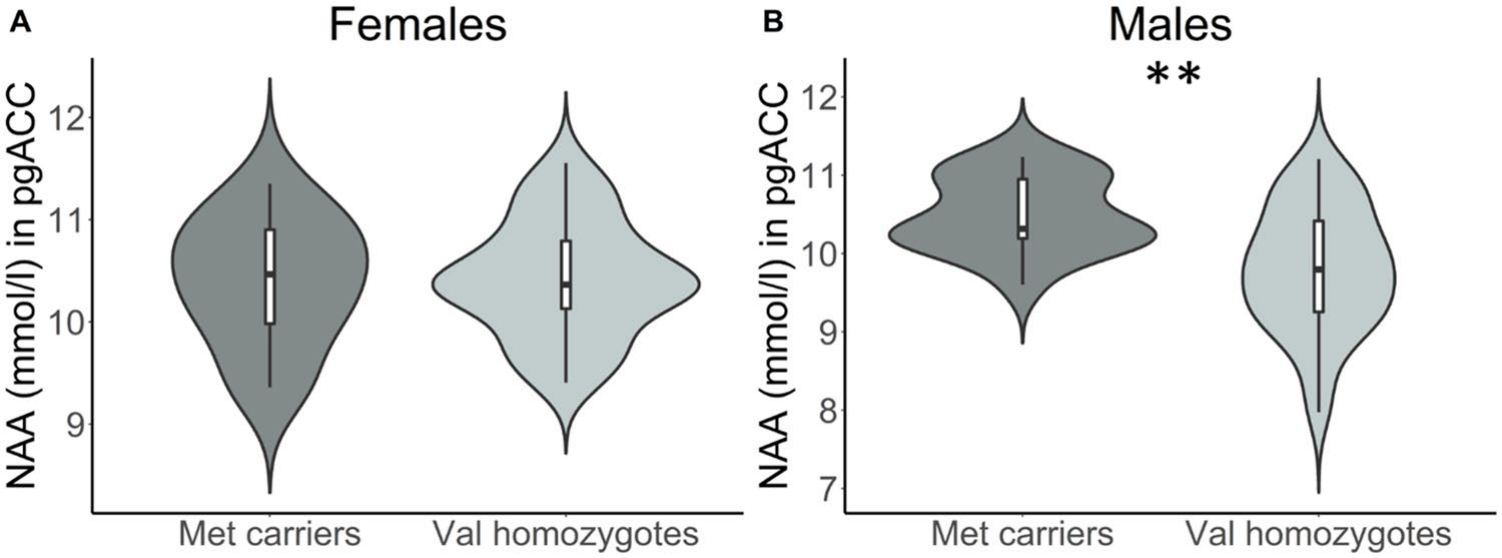
Gene-gender interaction on NAA. Results of follow-up linear models for female (**A**) and male (**B**) participants in cohort 2. Metabolite concentrations are corrected for the influence of age. mmol = millimolar. ** *P* < .01.

In the analysis of NAA, we found a significant main effect of gender, *F*(1,90) = 7.807, *P* = 0.006, *η*^2^_partial_ = 0.068. To further explore this, we included the interaction term BDNF*gender in the linear model. This resulted in a no longer statistically significant main effect of *BDNF* group (*F*(1,89) = 0.012, *P* = 0.912) but a significant interaction effect, *F*(1,89) = 5.598, *P* = 0.020. When followed up with separate linear models for each gender, men showed a significant main effect of genotype, *F*(1,52) = 10.585, *P* = 0.002 (Fig. 3), such that male Met carriers had higher NAA (M = 10.492, SD = 0.480) than Val homozygotes (M = 9.797, SD = 0.791). For female subjects separately, there was no significant main effect of genotype, *F*(1,36) = 0.018, *P* = 0.893.

## Discussion

In two independent samples of healthy participants, we investigated the effects of the *BDNF* Val66Met polymorphism on measures of excitatory neurotransmission in relation to neuronal integrity in the pgACC, a region of key importance in the pathophysiology of mood disorders such as MDD. The results from the independent cohorts provide converging evidence for a reduction of Glx/NAA in Met carriers. This is in line with our hypotheses. Follow-up analyses of the metabolites comprising the ratio revealed that, contrary to our primary expectation, this effect is due to an increase in NAA rather than a decrease in Glx. To our knowledge, this is the first study assessing the influence of this SNP on glutamatergic and NAA metabolism in the pgACC. These findings were strengthened by the fact that the two independent cohorts reported here were measured at different field strengths (3 T vs 7 T), used different MRS acquisition schemes (PRESS vs STEAM), and used male-only versus male and female participants, respectively.

Given the shared metabolic pathways of NAA and Glu, the ratio of Glx/NAA may be a more sensitive marker of (dys)regulation in related metabolites. NAA is highly abundant in the brain, where it is predominantly found in neurons. It is most highly concentrated in the mitochondria of pyramidal glutamatergic neurons^48^, has a role in energy metabolism, osmoregularity, and myelin formation, and is frequently described as a marker of neuronal viability^23^. NAA synthesis is coupled to the capacity of mitochondria for ATP synthesis^49,50^. Glu is not only the main excitatory neurotransmitter, it has roles in energy metabolism as well^24,51^. NAA and Glu are linked mainly through the tricarboxylic acid (TCA) and glutamate-glutamine cycles^24^. Although the precise role of NAA in the brain is debated, one proposal suggests that NAA serves as a reservoir for glutamate synthesis^30^. This assumption is based on a cycle converting NAA into aspartate and acetate by aspartoacylase in astrocytes and oligodendrocytes. Aspartate and acetate are then converted to oxaloacetate and acetyl CoA, important constituents of the TCA cycle. In the mitochondria, these intermediates are combined to form citrate. Citrate is oxidized to alpha-ketoglutarate, which is then converted to glutamate via transamination. Glutamate can also re-enter the TCA cycle for ATP production^24^.

Consistent with this metabolic cycle, some studies have shown correlations between MRS measures of local NAA and glutamatergic metabolites in healthy controls, that were disrupted in patients. A ^13^C-MRS study showed that the rate of glutamate labeling from ^13^C-glucose infusions was tightly coupled with NAA synthesis^52^. In healthy participants, studies have found positive correlations between NAA and Glx in the cerebellum^31^, hippocampus^33^, pgACC^32^ but conflicting results for the dorsal ACC^31,53^. In MDD patients, pgACC NAA and Glx were more strongly correlated in patients compared to healthy control participants^32^. Patients with schizophrenia, in contrast, showed a decoupling of NAA and Glx in the hippocampus^33^ and dorsolateral PFC^53^. In summary, the equilibrium between glutamate and NAA related metabolism may be disturbed in different classes of psychiatric disorders.

Our finding of reduced Glx/NAA in Met carriers is particularly interesting, since studies investigating psychiatric and neurological disorders associated with tissue damage such as multiple sclerosis (MS) have reported an overall increase of Glx/NAA or Glu/NAA compared to healthy participants^54^. Patients with MS had elevated ratios of glutamatergic metabolites to NAA in white matter compared to healthy controls which correlated with symptom severity^54^ and longitudinal brain volume loss^55^. While *BDNF* Val66Met has not been associated with disease risk or severity in MS^56–58^, a protective role for the Met allele has been suggested with respect to preserving grey matter integrity^59,60^ and maintaining memory network function^61,62^ in MS patients. This same increase is also seen in patients with disorders linked to decreased prefrontal and hippocampal volume such as schizophrenia and MDD when compared to healthy control participants^33,63^. In the hippocampus of patients with schizophrenia^33^ and post-traumatic stress disorder^64^, Glx/NAA and Glu/NAA respectively were elevated, which could be due to excitotoxic effects of excessive glutamate. In the ACC, NAA/Glx was reduced in patients with bipolar disorder compared to healthy controls and the ratio was normalized after a lamotrigine treatment^65^. In depressed youth with current suicidal ideation, pgACC Glx/NAA was higher compared to depressed youth without current suicidal ideation and compared to healthy control participants^63^. Similarly, another study found that medial prefrontal Glx/NAA (but not Glu/NAA) was higher in patients with MDD compared to healthy participants^66^. The Val66Met polymorphism has been linked to an increased risk for the development of MDD^34^ and schizophrenia^8,35^, although there is conflicting evidence^36^. Some studies report that the Met allele is a protective factor against development of schizophrenia^37^. Others report that Val66Met is not a risk factor for developing MDD^38^ or only a risk factor in males^67^. In general, an increased ratio of glutamatergic metabolites to NAA is associated with poorer mental health outcomes. A decrease in Glx/NAA in Met carriers hints at a potential protective effect of this genotype, but the mechanisms through which this may arise are unclear. One rather straight-forward explanation might be a higher overall availability of BDNF in Met carriers, which has been reported for BDNF serum levels and suggested to reflect a compensatory increase in constitutive secretion of BDNF^68^. Alternatively, or more likely additionally, a potential modulation of BDNF signaling via the p75 neurotrophin receptor by *BDNF* Val66Met might be considered a candidate mechanism^5^. The p75 receptor has been implicated in neuroinflammatory processes^69–71^, and a recent study suggests that higher serum BDNF 66Met carriers may be associated with lower concentrations of the pro-inflammatory cytokine TNFa^72^. However, the sample size in that study was relatively small, and future studies will be required to follow up on this intriguing possibility.

To further investigate the effects of Val66Met on pgACC metabolism, we capitalized on the better signal dispersion at 7 T by computing absolute quantified values for Glu, Gln and NAA. In contrast to our hypothesis, reduced Glx/NAA in Met carriers was unlikely to be due to glutamatergic differences, as we found no significant difference in glutamatergic (Glu or Gln) levels between the two genotypes. This is striking, because the Val66Met SNP is associated with reduced BDNF signaling^11^ and reduced glutamatergic neurotransmission^17,18^, but on the other hand at any point in time, a proportion of glutamate serves metabolic roles and it appears that vesicular, neurotransmitter glutamate cannot be detected with MRS^73,74^. Subtle changes in neurotransmitter glutamate could therefore have gone undetected in our sample. Another possible reason may be that there is a dose-dependent effect of *BDNF* Val66Met on glutamatergic measures. Reduced Glx was previously reported in the hippocampus and posterior medial frontal cortex^26^ but only in Met homozygotes. Only one participant in cohort 1 carried two Met alleles, and no participants in cohort 2 were Met homozygotes. The relative rarity of homozygotic Met carriers may have impaired the detection of possibly subtler, dose-dependent effects of the Met-allele on the glutamatergic metabolites^6,75,76^. Moreover, both cohorts were young healthy participants and it may be that the effects of Val66Met are age-related and more relevant in older age^77,78^.

An intriguing finding is that of a significant increase in NAA in Met carriers. Apart from reduced spine density and other morphological abnormalities, Met carriers (healthy controls as well as patients) were previously shown to have reduced NAA in the hippocampal formation^6,26,27^ and lacking increase after training for a difficult task^79^. Given the evidence for reduced neuronal integrity in Met carriers in the hippocampus, our finding of increased NAA in Met carriers in the pgACC is particularly remarkable. However, an earlier study at 3 T similarly reported an NAA increase in Val66Met Met carriers in the ACC^28^. Our result therefore strengthens their finding and speaks for region-dependent effects. Gallinat et al. suggest that higher pgACC NAA may protect from developing psychiatric illnesses such as schizophrenia and bipolar disorder^28^. These disorders are associated with an altered ratio of Glx/NAA^33,65^ as well as a decoupling between glutamatergic metabolites and NAA^33,53^. In MDD and schizophrenia, research suggests a decrease in NAA concentrations as the disease progresses^29,80–84^. Taking into account the aforementioned shared metabolic pathways of glutamate and NAA, a relative increase of NAA in the pgACC of Met carriers may serve to readily and adaptively provide glutamate when necessary to maintain normal neuronal functioning^29,50,85^. The molecular pathways or gene–gene interactions that link the effects of Val66Met on BDNF release to an increase in NAA remain to be investigated.

Meta-analytic evidence suggests that the Met allele confers increased risk for developing MDD^34^, specifically in males^67^. One explanation for discrepant results may be related to gene-environment interactions, as another meta-analysis found that the association between the Met allele and depression risk might be primarily found in individuals with high levels of life stress or childhood adversity^86^. Although Met carriers and Met/Met homozygotes showed larger cortisol responses to acute stress^87,88^, gender specific findings seem to be more controversial as some studies reported an attenuated cortisol response in male^89,90^ and others in female^91^ Met carriers. Cognitive performance was reported to improve from physical activity in male Val homozygotes only^92^, while the decline in executive functioning and processing speed over time is lowest in female Met carriers^93^. In an exploratory analysis, we found an interaction between genotype and gender such that male but not female Met carriers showed increased NAA in the pgACC. In healthy subjects, pgACC NAA has been shown to not differ significantly between males and females^47^. Assuming male Met carriers are more likely to develop MDD, we may have investigated a sample of healthy, resilient males with compensatory higher NAA levels. Further, a compensatory increase in NAA in Met carriers may not be necessary in females, as ovarian hormones such as estrogen increase BDNF mRNA levels in the cingulate cortex in rodents^94^. Hormone cycle specific explanations need further investigation, as we did not control for that in the current study.

Whereas earlier studies reported differences in the ratio of gray matter (GM) volume between Met carriers and Val homozygotes, we did not observe a significant difference in GM tissue proportion in the pgACC voxel in neither of the two cohorts. In previous studies, Met carriers showed reduced GM volume in the hippocampus^22,95^, and dorsolateral prefrontal areas^22,96,97^ when compared to Val homozygotes. For the ACC, there is not much evidence for such a reduction. Montag et al. found greater GM volume in the ACC in Val homozygotes compared to Met carriers, but lowest ACC GM volume in carriers of two different polymorphisms^98^. *BDNF* Val66Met carriers were also shown to have greater GM volumes in temporal and superior frontal areas^99^. GM volume differences of the Val66Met polymorphism seem to be region specific. In addition, the relative increase in pgACC NAA in Met carriers may play a role in prevention of cortical GM reduction resulting from excitotoxic effects. The absence of a difference in GM proportion between the two genotypes is therefore not surprising.

Although our study has many strengths, it should be considered in the context of some limitations. Due to limited previous literature and reported effect sizes, we did not calculate a priori sample sizes. Post hoc power calculation showed that for Cohort 1 power was 82%, and for Cohort 2 61%. For cohort 1, we did not perform an absolute quantification of NAA due to lower signal dispersion at 3 T. Therefore, we could not check for the effect of NAA we observed in the 7 T sample. Due to the specification to optimally resolve Gln from Glu in the study with 7 T, the contribution of Gln to the measured Glx may not be substantially suppressed as the 3 T PRESS (80 ms) sequence. Our results have to be interpreted with caution. As the gender groups in our 7 T sample were not balanced, we cannot draw definitive conclusions about a male-specific effect of *BDNF* Val66Met on pgACC NAA levels. This should be addressed in future studies. Further, we did not include the hippocampus as a control region since our hypothesis concentrated on the pgACC. Further, the cohorts differed in the GM ratio, with lower values in cohort 2. This might be related to the better contrast due to the higher spatial resolution in the 7 T images. It was shown previously that more accurate segmentation at higher field strength led to lower estimates of cortical thickness^100^. Last, we report measures of NAA and not the commonly used tNAA (total NAA; the sum of NAA and NAAG). Glx/tNAA and Glx/NAA were strongly correlated at 3 T (*r*(25) = 0.954, *P* < 0.001, 95% CI = [0.900, 0.980]) and 7 T (*r*(96) = 0.903, *P* =  < 0.001, 95% CI = [0.860, 0.934]). Therefore, we have repeated these with tNAA instead of NAA. At 3 T, the effect of *BDNF* Val66Met genotype remained significant (*F*(22) = 6.150, *P* = 0.021, *η*^2^_partial_ = 0.221). At 7 T, the main effect of *BDNF* genotype was no longer significant, *F*(92) = 2.839, *P* = 0.100. Strikingly, there was also no longer a significant main effect of gender, *F*(92) = 2.036, *P* = 0.157. In the analysis of absolute levels of tNAA, there were no significant main effects of *BDNF* genotype (*F*(90) = 2.746, *P* = 0.101) nor of gender (*F*(90) = 3.413, *P* = 0.068). Taken together, these findings suggest that the better separation of NAA and NAAG at 7 T enables us to identify an effect of *BDNF* Val66Met that can be attributed to NAA and not to glutamate-related metabolites.

To sum up, we found an altered ratio of Glx/NAA in carriers of the *BDNF* Val66Met polymorphism in two independent samples. Although many psychiatric and neurological disorders are associated with an increase in Glx/NAA, Met carriers of the *BDNF* SNP showed a decrease in this ratio. Against our expectations, follow up analyses suggest that glutamatergic metabolism is not affected by the polymorphism. Instead, Met carriers showed a relative increase in NAA in the pgACC. Future research should test whether this increase protects the region from effects of psychiatric disorders such as schizophrenia or MDD.

## Methods

### Participants

We investigated two independent samples of healthy subjects. Cohort 1, measured at 3 T, consisted of 30 healthy men (mean age = 29.00 ± 5.55). Cohort 2 measured at 7 T comprised 98 healthy participants (mean age = 26.64 ± 6.38, 40 women). Some participants of cohort 2 were recruited as the control group of a patient study. This cohort of healthy subjects was previously used in a published analysis on the association between glutamatergic disbalance and harm avoidance^101^. All participants were screened for prior and current neurological or psychiatric illnesses using the German version 5.0.0 of the Mini International Neuropsychiatric Interview (M.I.N.I.)^102^. All study procedures obtained the approval of the ethics committee of the University of Magdeburg, Faculty of Medicine, and conformed to the Declaration of Helsinki. All participants received detailed information on the study and provided written informed consent to participate.

### Genotyping

Whole–blood samples were collected from participants in EDTA–coated tubes (BD Vacutainer, K3E, 7.2 mg. REF 368,884) and stored at 4 °C. Genomic DNA was extracted from whole blood using the GeneMole automated system (Mole Genetics AS, Lysaker, Norway) according to the manufacturer’s protocol. Genotyping was performed using PCR followed by allele–specific restriction analysis. Briefly, the DNA fragment on chromosome 11p14.1 containing the *BDNF* val66met polymorphism (NCBI accession number: rs6265) was amplified using the primers *BDNF_rs6265_f* (forward): 5′- GCA TCC CGG TGA AAG AAA GCC CTA AC-3′ and *BDNF_rs6265_r* (reverse): 5′- GCC CCT GCA GCC TTC TTT TGT GTA AC-3′, and standard Taq polymerase (Qiagen). The resulting PCR products were digested with the PmaCI isoschizomer Eco721 (ThermoFisher Scientific), yielding two allele-specific amplicons (398 + 278 bp) for the more common Val allele, and the entire region (676 bp) for the less common Met allele. DNA fragments were separated on a 2.5% agarose gel stained with Midori Green (Biozym Scientific, Hessisch Oldendorf, Germany) and visualized under UV light.

### Magnetic resonance spectroscopy data acquisition and analysis

#### Cohort 1

**Figure 4:**
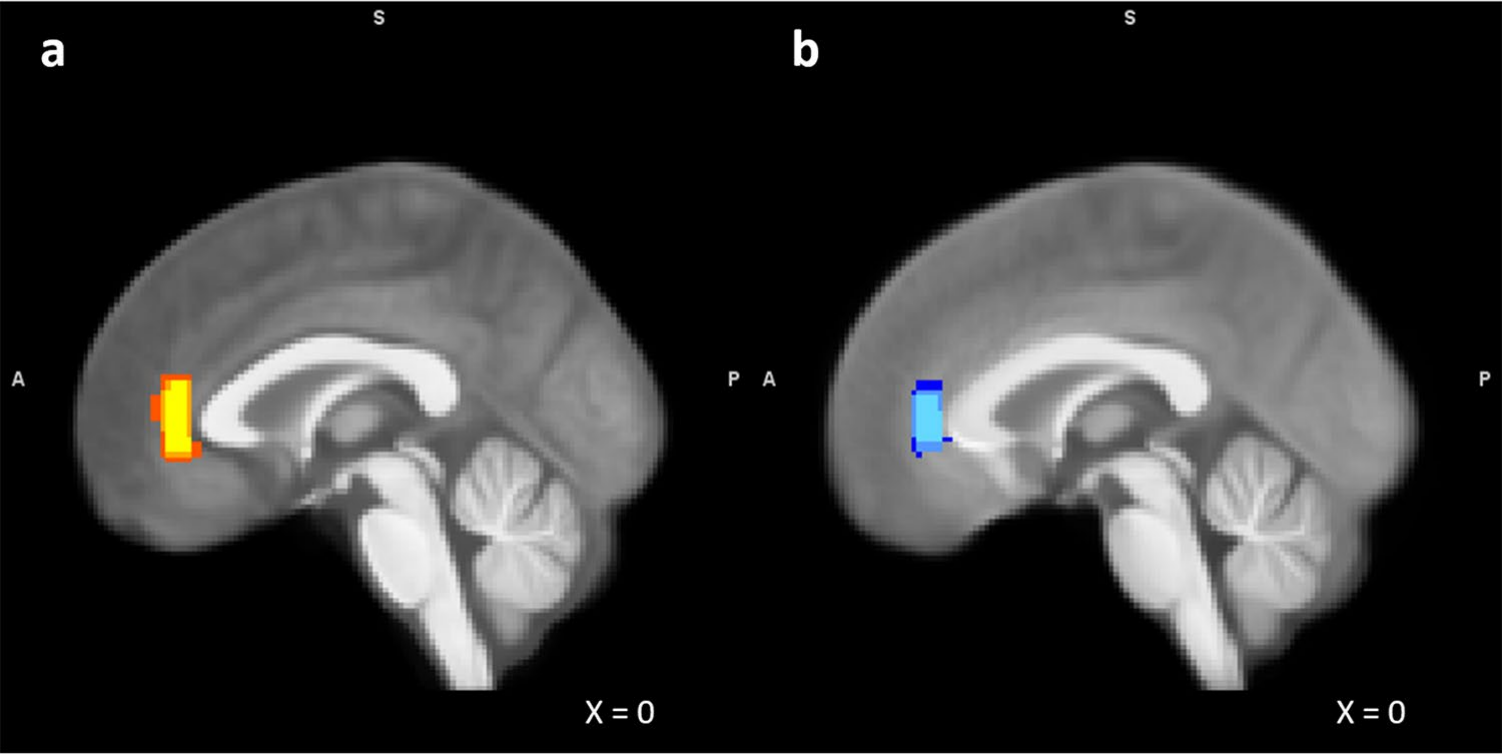
Overlap of individual MRS voxel placements in cohort 1 (**a**) and cohort 2 (**b**) overlaid on the mean T1-weighted image of participants in each respective cohort. A: 3 T sample (n = 27). Red = 25% overlap. Orange = 50% overlap. Yellow = 75% overlap. B: 7 T sample (n = 98). Dark blue = 25% overlap. Blue = 50% overlap. Light blue = 75% overlap.

With cohort 1, we conducted all measurements using a 3 T MAGNETOM Trio MRI scanner with an 8-channel head coil (Siemens, Erlangen, Germany). Prior to MRS measurements, we acquired a high-resolution structural magnetization-prepared rapid gradient-echo (MPRAGE) T1-weighted scan (echo time (TE) = 4.77 ms, repetition time (TR) = 2.5 s, inversion time (TI) = 1.1 s, flip angle = 7°, bandwidth = 140 Hz/pixel, acquisition matrix = 256 × 256 × 192, isotropic voxel size = 1.0 mm^3^). These anatomical scans were used for accurate placement of the pgACC voxel according to an established protocol of anatomical landmarks, as described by Dou et al.^103^. MRS spectra were acquired in the bilateral pgACC (20 × 10 × 20 mm^3^) (Fig. 4a). We centered voxels on the sagittal midline to ensure maximal coverage of relevant gray matter areas. To optimize B_0_ homogeneity, we used automatic shim routines. This procedure took 1–5 min, varying from participant to participant. A point-resolved spectroscopy (PRESS) sequence was used with the following parameters: TE = 80 ms, TR = 2 s, 256 averages, band width = 1200 Hz, acquisition time for one image = 853 ms^104^.

**Figure 5:**
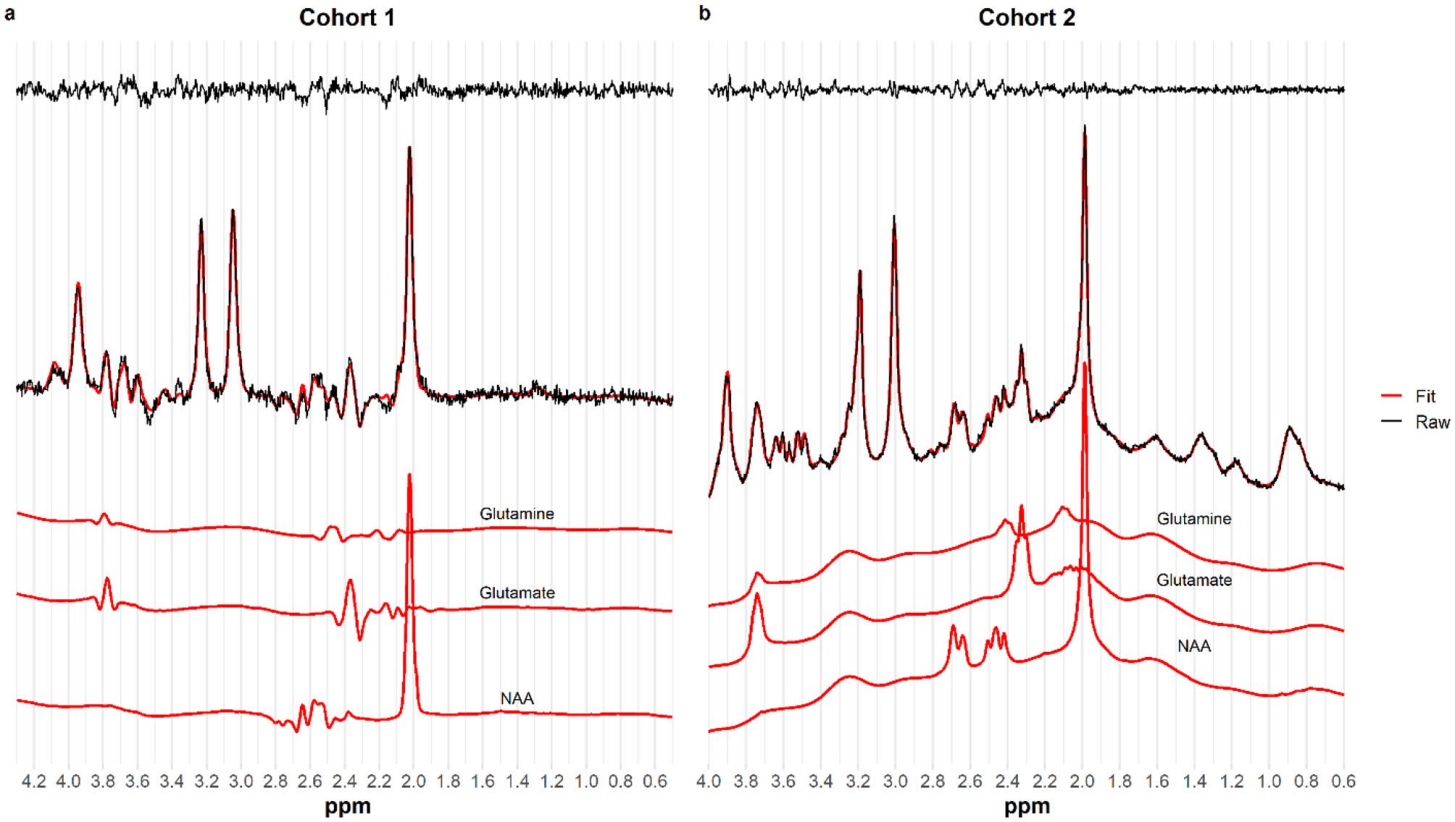
Example spectra. A: 3 T (PRESS), B: 7 T (STEAM). Residuals are shown at the top of both panels.

The spectra were analyzed using LCModel version 6.1.0^105^. We used a measured basis set that included sixteen metabolites: Creatine (Cr), Glutamate (Glu), Myo-Inositol, Lactate, N-Acetylaspartate (NAA), Phosphocholine (PCh), Taurine, Aspartate, γ–Aminobutyric acid (GABA), Glutamine (Gln), Glucose (Glc), Alanine, N–acetylaspartylglutamate (NAAG), Phosphocreatine (PCr), Guanidinoacetate , and Glycerophosphocholine. The default simulated macromolecules were included in the modelling. The analysis window was set to range from 4.3 ppm to 0.5 ppm. Eddy current correction was performed based on the water signal (LCModel parameter DOECC = T) and water suppression was performed (DOWS = T). The attenuation factors of water (ATTH2O) and metabolites (ATTMET) were assumed to be 0.2905 and 0.4079, respectively. The standard deviation of the expected value for zero-order phase correction (SDDEGZ) and first-order phase correction (SDDEGP) were set to 0.5 and 0.2, respectively. For our analyses, we used a joint measurement of glutamate and glutamine (Glx), expressed as a ratio to NAA. We used conventional criteria to ensure sufficient spectral quality. That is, metabolite values with Cramér Rao lower bound (CRLB) estimates of fitting error > 20%^106^, FWHM > 12 Hz, or SNR < 8 were excluded from further analyses. An exemplary spectrum is shown in Fig. 5a.

#### Cohort 2

With cohort 2, we acquired the ultra-high field data using a 7 T MAGNETOM scanner equipped with a 32-channel head coil (Siemens, Erlangen, Germany). Before MRS measurements, we acquired an MPRAGE T1-weighted scan (TE = 2.73 ms, TR = 2.3 s, TI = 1.05 s, flip angle = 5°, bandwidth = 150 Hz/pixel, acquisition matrix = 256 × 256 × 224, isotropic voxel size = 0.8 mm). MRS voxel placement in the pgACC conformed with procedures described for cohort 1 (20 × 10 × 15 mm^3^) (Fig. 4b). Automatic shim routines were performed before acquiring MRS spectra to homogenize the B_0_ field. We used a STEAM sequence with the following parameters: TE = 20 ms, TR = 3 s, TM = 10 ms, 128 averages, bandwidth = 2800 Hz, and acquisition time for one image = 731 ms. For eddy current correction and absolute quantification of metabolite values, we acquired a non-water-suppressed reference scan with one instance.

The data was fitted using LCModel version 6.3.0^105,107^. We used a measured basis set that included Creatine (Cr), Glutamate (Glu), Myo-Inositol, Lactate, N-acetylaspartate (NAA), Phosphocholine (PCh), Taurine, Aspartate, γ-Aminobutyric acid (GABA), Glutamine (Gln), Glucose (Glc), Alanine, N-acetylaspartyl-glutamate (NAAG), Phosphocreatine (PCr), Scyllo-inositol, Acetate, Succinate, Phosphorylethanolamine, Glutathione (GSH), Citrate, and Glycerophosphocholine. The default simulated macromolecules were included in the modelling. The analysis window was set to range from 4.0 ppm to 0.6 ppm. Eddy current correction was performed based on the water signal (LCModel parameter DOECC = T) and water suppression was performed (DOWS = T). The attenuation factors of water (ATTH2O) and metabolites (ATTMET) were assumed to be 0.67 and 0.69, respectively. The chemical shift of the singlet used for scaling (Cr) was set to 3.0 ppm. To account for uncertainty in the referencing between in vitro (the simulated basis set) and in vivo measurements, the default SD of shift (DESDSH) and the expectation of 1/T2 (DEEXT2) were set to 0.01 and 12.0, respectively. Additional changes in the SD of shift (ALSDSH) from the new default SDSH were applied for NAA (0.004), NAAG (0.004), Glc (0.025), and PCh (0.025). Lactate, Scyllo-inositol and Acetate were omitted (CHOMIT) from the basis set used for fitting. Figure 5b shows an exemplary spectrum. Metabolite values were considered of insufficient quality if SNR < 20, FWHM > 24 Hz, or CRLB < 20%. Metabolite values were calculated as the ratio of Glx to NAA^108^. In exploratory analyses, we capitalized on the increased signal dispersion that ultra-high field provides, and calculated the absolute concentrations of Glu, Gln, and NAA. During the absolute quantification procedure, corrections were applied for the proportions of gray matter, white matter, and cerebrospinal fluid in each MRS voxel. For more details on the absolute quantification procedure, see Giapitzakis et al.^109^.

#### Segmentation of T1 images

To account for differences in tissue composition within each participant’s MRS voxel, we segmented each individual MRS voxel using voxel-based morphometry (VBM) as implemented in the CAT12 toolbox (www.neuro.uni-jena.de/vbm/) and expressed the gray matter (GM) proportion in the voxel as the segmented GM within the voxel divided by the total volume of the voxel.

### Statistical analysis

We performed all statistical analyses in R 3.4.4 (R Core Team, 2018) with RStudio IDE Version 1.1.383 (RStudio Team, 2015). Before the actual statistical analyses, we verified that the allele frequency distribution of the rs6265 was at Hardy–Weinberg equilibrium.

After excluding datasets that did not meet our MRS quality assessment (see above), the cohort 1 sample used in subsequent analyses consisted of 27 participants (all men, age = 29.78 ± 5.29 years, Met carriers/Val homozygotes: 10/17), and the cohort 2 sample consisted of 98 participants (40 women, age = 26.64 ± 6.38, Met carriers/Val homozygotes: 30/68), unless otherwise specified in the exploratory analyses.

Differences in covariates (age, gender, GM proportion) were analyzed using Welch independent sample t-tests and Chi-square tests.

To investigate the effect of *BDNF* genotype (Val homozygotes vs. Met carriers) on pgACC Glx/NAA values at 3 T and at 7 T, we computed a linear regression model with Glx/NAA as the dependent variable and *BDNF* genotype as fixed factor. As covariates, we included gender (only for cohort 2 analyses, as cohort 1 included only male participants), age, and proportion of GM determined with VBM. The choice of our nuisance covariates was based on previous work suggesting that metabolite concentrations may vary as a function of age^110,111^, sex^47,112^, and voxel tissue composition^113^. We assessed normal distribution of residuals using Shapiro–Wilk tests of normality, and homogeneity of variances using Levene’s test. Outlier detection and removal was done using boxplots (values more than 1.5* interquartile range (IQR) below the first quartile or above the third quartile were considered outliers)^114^. To assess the directionality of significant main effects identified by the linear model, we performed Welch’s t-tests for unequal variances, testing for differences of Glx and NAA levels between *BDNF* genotype groups.

Because we addressed the same question in cohort 1 and cohort 2, and study samples were independent, we used Fisher’s method of combining *P*-values^115^ to test the global null hypothesis that *BDNF* genotype, corrected for covariates, has no effect on pgACC Glx/NAA levels.

Using the 7 T data from cohort 2, we performed post hoc analyses of Glu, Gln, and NAA, using the same statistical approach as in the analyses of Glx/NAA. The goal of these additional analyses was to investigate the metabolite specificity of our findings. Based on post hoc results, we performed exploratory analyses to investigate potential effects of gender in our data.

The significance level was set at ⍺ = 0.05. To control for multiple comparisons in our *post-hoc* analyses, we used a Bonferroni correction for the three metabolites (effective threshold: ⍺ = 0.017).
